# Pulmonary Immune Cell Landscape Altered by Exposure to HIV, *Schistosoma* and Their Combination

**DOI:** 10.3390/ijms27125426

**Published:** 2026-06-16

**Authors:** Daniel Morales-Cano, Sandra Medrano-Garcia, Bianca Barreira, Ana Hernández-García, Rahul Kumar, Brian B. Graham, Rajkumar Savai, Soni Savai Pullamsetti, Francisco Perez-Vizcaino, Ghazwan Butrous, Angel Cogolludo, Edgar Fernández-Malavé

**Affiliations:** 1 Department of Pharmacology and Toxicology, School of Medicine, Universidad Complutense de Madrid, Instituto de Investigación Sanitaria Gregorio Marañón, 28040 Madrid, Spain; biancabarreira@med.ucm.es (B.B.); anaher10@ucm.es (A.H.-G.);; 2 Centro de Investigación Biomédica en Red Enfermedades Respiratorias, (CIBERES), 28029 Madrid, Spain; 3 Department of Immunology, Ophthalmology and ENT, School of Medicine, Universidad Complutense de Madrid, Instituto de Investigación Sanitaria Hospital 12 de Octubre (imas12), 28040 Madrid, Spain; samedran@ucm.es (S.M.-G.); edfernan@med.ucm.es (E.F.-M.); 4 Institute for Lung Health (ILH), Justus Liebig University, 35305 Giessen, Germany; rajkumar.savai@mpi-bn.mpg.de (R.S.); soni.pullamsetti@mpi-bn.mpg.de (S.S.P.); 5 Department of Translational Immunology, Genentech, Inc., 1 DNA Way, South San Francisco, CA 94080, USA; kumar.rahul.rk1@gene.com; 6 Department of Medicine, University of California, San Francisco, CA 94143, USA; brian.graham@ucsf.edu; 7 Max Planck Institute for Heart and Lung Research, Member of the German Center for Lung Research (DZL), Member of the Cardio-Pulmonary Institute (CPI), 61231 Bad Nauheim, Germany; 8 Cardiopulmonary Sciences, University of Kent Canterbury, Kent CT2 7NZ, UK; g.butrous@kent.ac.uk

**Keywords:** HIV proteins, lungs, *Schistosoma*, immune cells, pulmonary arterial hypertension

## Abstract

Local immune cell activation and vascular remodelling are characteristic pathogenic features of pulmonary arterial hypertension (PAH). HIV and schistosome infections have been individually associated with PAH. However, whether co-infection with these pathogens has a distinct impact on the development of pulmonary vascular disease remains poorly understood, partly due to the lack of experimental animal models. In a novel non-infectious model of HIV and *Schistosoma* pulmonary co-exposure based on lung embolisation of *S. mansoni* eggs in HIV-transgenic (HIV) mice, we previously reported exacerbated endothelial remodelling and dysfunction, along with increased pulmonary arterial pressure; which were associated with a unique profile of pro-inflammatory cytokines in the lung. In the present study, we used flow cytometric analysis of isolated lung leukocytes and immunofluorescence staining to characterise the pulmonary immune cell landscape associated with individual or combined exposure to HIV and schistosome. Compared with mice exposed to HIV (untreated HIV mice) or schistosome (egg-treated wild-type mice), co-exposed (egg-treated HIV mice) animals showed significantly increased numbers of interstitial and alveolar macrophages, patrolling-type monocytes, NKT and γδ T cells, and reduced CD8^+^ αβ T cells. Other lung immune cells, including inflammatory-type monocytes, eosinophils/neutrophils, dendritic cells, CD4^+^ αβ T cells, NK cells and B cells were not significantly affected in the co-exposure condition. Taken together, these results show for the first time that combined pulmonary exposure to HIV and *Schistosoma*, as it may occur in co-infected individuals, alters the local immune cell landscape in a manner distinct from that of individual exposure. Furthermore, these findings may contribute to a better understanding of the complex inflammatory processes involved in the pathogenesis of PAH, thereby supporting the development of therapies targeting pathogenic immune cells in pulmonary vascular disease associated with HIV and *Schistosoma* co-morbidity.

## 1. Introduction

By the end of 2024, an estimated 40.8 million people were living with HIV worldwide. Although HIV infection is quite prevalent in developed countries, the highest burden remains in Sub-Saharan Africa. People living with HIV are treated with antiretroviral therapy (ART); however, it remains a lifelong treatment with no definitive cure. Individuals who adhere to ART typically show an apparent clearance of the virus from peripheral blood shortly after starting therapy [1]. However, viral particles can be detected after discontinuing ART, suggesting that viral transcription persists in tissue reservoirs despite treatment [2,3,4].

Since the discovery of HIV, pulmonary complications have been a major cause of morbidity and mortality [5]. ART has increased life expectancy and altered the spectrum of infection-related pulmonary complications. Nevertheless, non-infectious HIV-associated pulmonary diseases, including chronic obstructive pulmonary disease, asthma, lung cancer, and pulmonary vascular disorders—particularly pulmonary arterial hypertension (PAH)—remain a significant cause of morbidity and mortality [1,6].

PAH is a progressive disorder characterised by pulmonary arterial remodelling, which leads to elevated pulmonary artery pressure and increased pulmonary vascular resistance, ultimately resulting in right ventricular failure and death if left untreated [7,8]. In addition to idiopathic and heritable forms, infectious diseases—particularly those caused by HIV and *Schistosoma* species—are two of the leading causes of PAH worldwide [6,9,10,11]. Notably, approximately 200 million individuals infected with schistosomes live in African countries with widespread HIV-1 epidemics. It is estimated that, in Africa alone, co-infection with HIV and schistosomes affects around 6 million people [12,13]. Therefore, this epidemiological overlap with HIV and *Schistosoma* represents a plausible and understudied scenario that may contribute to the development of severe and rapidly progressive PAH [14].

The relevance of immune cells to pulmonary hypertension, particularly PAH, has been increasingly recognised [15]. HIV infection can affect both innate and acquired immune function in the lung [6,16,17], even in people treated with ART [1]. Schistosome infection also causes pulmonary immune cell activation and associated pathology [18,19]. In contrast, the impact of HIV and schistosome co-infection on lung immune cells remains completely unknown.

To address this gap, we recently characterised the impact of HIV and *Schistosoma* co-exposure on pulmonary circulation in a non-infectious mouse model [20]. Specifically, HIV-1 (Tg26)-transgenic mice and their wild-type counterparts were treated with *S. mansoni* eggs according to a standard protocol [20,21,22] that induces a type-2 inflammation response in the lung, pulmonary granuloma formation and pulmonary vascular remodelling. Our data revealed that both HIV and schistosome impaired pulmonary vascular endothelial function, and their combination promoted aberrant endothelial remodelling and dysfunction associated with aggravated pulmonary vascular pathology [20]. These pulmonary vascular abnormalities were associated with unique changes in the expression of pro-inflammatory and profibrotic cytokines, but the impact of persistent co-exposure to HIV and schistosome on lung immune cell populations was not evaluated. Therefore, herein we have assessed the profile of pulmonary immune cells in mice exposed to HIV and schistosome, individually or in combination. Our data reveal specific and previously unrecognised alterations in the immune cell landscape that occurs in the lung following concurrent exposure to HIV and *Schistosoma*, providing new insight into how co-infection may contribute to pulmonary vascular disease.

## 2. Results

### 2.1. Lung Expression of Viral Proteins in HIV-Transgenic Mice Is Restricted to Leukocytes and Does Not Affect the Cellular Composition of Schistosome Egg-Induced Granulomas

**Figure 1:**
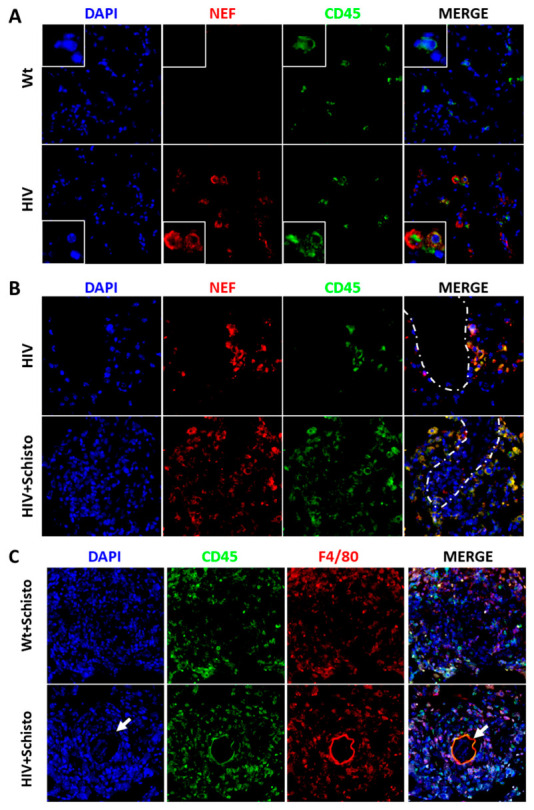
**Expression of HIV-1 NEF protein in the lungs of HIV mice.** (**A**) Representative images from IFM analysis of NEF expression in the lungs of Wt and HIV mice. (**B**) NEF expression around pulmonary vessels in untreated HIV mice or those treated with *Schistosoma* eggs. Vessels are outlined with a dotted line. CD45 staining was used to identify leukocytes and DAPI to stain cell nuclei. (**C**) F4/80^+^ cells in lung granulomas of schistosome egg-treated Wt and HIV mice. The arrow indicates the location of the *Schistosoma* egg.

In HIV (Tg26)-transgenic mice (hereafter referred to as “HIV” mice), viral transcripts are detectable in various tissues, including the lung [23]. Additionally, HIV-LTR mediates low-level transcription in immune cells such as monocytes, macrophages and lymphocytes [24]. To identify the cell types expressing viral proteins in the lungs of HIV-transgenic mice, tissue sections were analysed by immunofluorescence microscopy (IFM) after staining with antibodies against the HIV-1 protein Nef and the pan-leukocyte marker CD45, with wild-type (Wt) counterparts as controls. As shown in Figure 1A, Nef was undetected in Wt animals, as expected. In HIV mice, Nef was expressed exclusively in CD45^+^ cells and was not detected in lung parenchymal cells. Moreover, treatment of HIV mice with *S. mansoni* eggs resulted in an increased number of Nef-expressing leukocytes in the lungs from HIV mice (Figure 1B).

Schistosome infection is characterised by the formation of multicellular granulomas around trapped eggs in various tissues, including the lungs [25]. To assess the cellular composition of granulomas, lung sections from egg-treated Wt and HIV mice were examined by IFM (Figure 1C). In both groups, most DAPI^+^ nuclei within the granulomas co-localised with the leukocyte marker CD45. Notably, a prominent accumulation of F4/80^+^ macrophages was observed immediately adjacent to the eggs—a feature more evident in HIV mice. A smaller population of CD45^+^ F4/80^−^ cells, presumably T cells and eosinophils [25], was identified at the granuloma periphery in both experimental groups.

### 2.2. Pulmonary Interstitial and Alveolar Macrophages Are Increased in Mice Co-Exposed to HIV and Schistosome

The different populations of pulmonary myeloid cells were characterised by multiparametric flow cytometry (Supplementary Figure S1).

Pulmonary macrophages can be identified by the surface expression of F4/80 and CD64. Within this population, two main subsets found in the lung are interstitial macrophages (IMs) and alveolar macrophages (AMs), distinguished by the presence or absence of surface CD11b, respectively [26].

**Figure 2:**
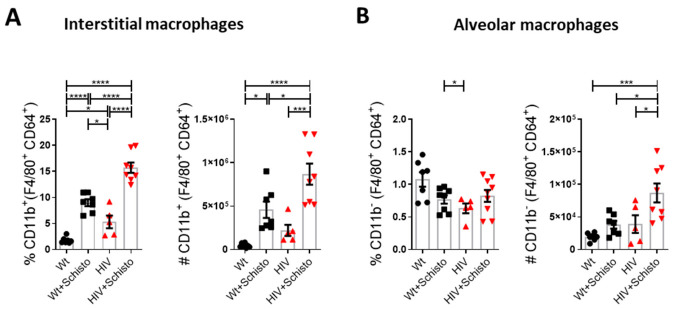
**Pulmonary macrophages in Wt and HIV mice exposed or unexposed to *Schistosoma* eggs.** Flow cytometric analysis of (**A**) interstitial (F4/80^+^ CD64^+^ CD11b^+^) and (**B**) alveolar (F4/80^+^ CD64^+^ CD11b^−^) macrophages in the lungs of Wt and HIV mice unexposed or exposed (+Schisto) to *Schistosoma* eggs (n = 5–8). Frequencies (%) and numbers (#) are shown. * *p* ≤ 0.05, *** *p* ≤ 0.001, **** *p* ≤ 0.0001 (one-way ANOVA Tukey).

Quantitative analysis showed that the frequency of IM—the subset that differentiates from infiltrating monocytes within the inflamed lung parenchyma—was significantly higher in HIV mice than in Wt controls, with a similar upward trend in cell numbers (Figure 2A). Although administration of schistosome eggs increased both the proportion and total number of IMs in all mice, this expansion was much more pronounced in the HIV group.

In contrast, the resident population of AM remained comparable between Wt and HIV mice. Following egg embolisation, only the HIV mice showed a significant increase in the AM population, although this response was less robust than that observed for IM (Figure 2B).

### 2.3. HIV and Schistosome Co-Exposure Promoted Expansion of Pulmonary Patrolling but Not Inflammatory Monocytes and Blunted the Granulocyte Response, While Having No Impact on Dendritic Cells

Monocytes are CD11b^+^ myeloid cells that can be further divided in two distinct functional populations based on the surface expression of the GR1 marker [27,28]. Hence, CD11b^+^ GR1^low^ monocytes are considered “patrolling” monocytes (which patrol blood vessels), while CD11b^+^ GR1^hi^ ones represent “inflammatory” monocytes, which are robustly recruited to sites of inflammation.

**Figure 3:**
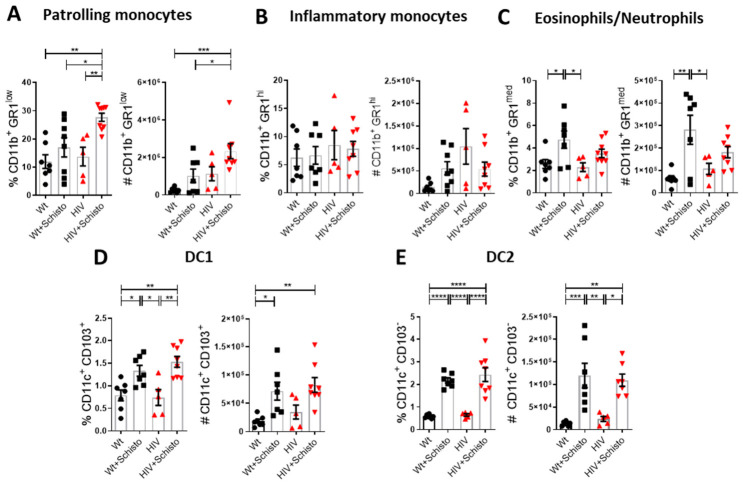
**Pulmonary monocytes, eosinophils/neutrophils and dendritic cells in Wt and HIV mice exposed or unexposed to Schistosoma eggs.** Flow cytometric analysis of (**A**) patrolling (CD11b^+^ GR1^low^) and (**B**) inflammatory (CD11b^+^ GR1^hi^) monocytes, (**C**) eosinophils/neutrophils (CD11b^+^ GR1^med^), (**D**) DC1 (CD11c^+^ CD103^+^) and (**E**) DC2 (CD11c^+^ CD103^−^) dendritic cells in the lung of Wt and HIV mice unexposed or exposed (+Schisto) to *Schistosoma* eggs (n = 5–8). Frequencies (%) and numbers (#) are shown. * *p* ≤ 0.05, ** *p* ≤ 0.01, *** *p* ≤ 0.001, **** *p* ≤ 0.0001 (one-way ANOVA Tukey).

Analysis of these populations revealed that patrolling monocytes were comparable between untreated Wt and HIV mice. However, following schistosome egg administration, this subset expanded significantly in HIV mice, a response that was notably more robust than in their Wt counterparts (Figure 3A). In contrast, the proportions and absolute numbers of inflammatory monocytes remained similar across all experimental groups, regardless of HIV status or egg treatment (Figure 3B).

We next assessed granulocyte populations (eosinophils and neutrophils) sharing a distinct surface CD11b^+^ GR1^med^ phenotype, which allows their discrimination from monocytes that express low or high levels of surface GR1 [27,28]. As shown in Figure 3C, the proportions and numbers of CD11b^+^ GR1^med^ cells were comparable in Wt and HIV mice. Upon parasite egg administration, Wt mice exhibited a significant increase in both the frequency and total count of these granulocytes. In contrast, this response was attenuated in HIV mice, showing only a minor upward trend.

Finally, we examined two pulmonary dendritic cell (DC) subsets defined by surface CD103 expression [29]. DC1 (CD11c^+^ CD103^+^) and DC2 (CD11c^+^ CD103^-^) were equally represented in the lungs of Wt and HIV mice. Following egg challenge, both DC1 and DC2 populations increased to a similar extent in both types of mice (Figure 3D,E).

### 2.4. Expansion of Pulmonary CD4^+^ and CD8^+^ αβ T-Cell Subsets by Schistosome Egg Treatment Is Reversed by HIV Exposure Only in the CD8^+^ Lineage

**Figure 4:**
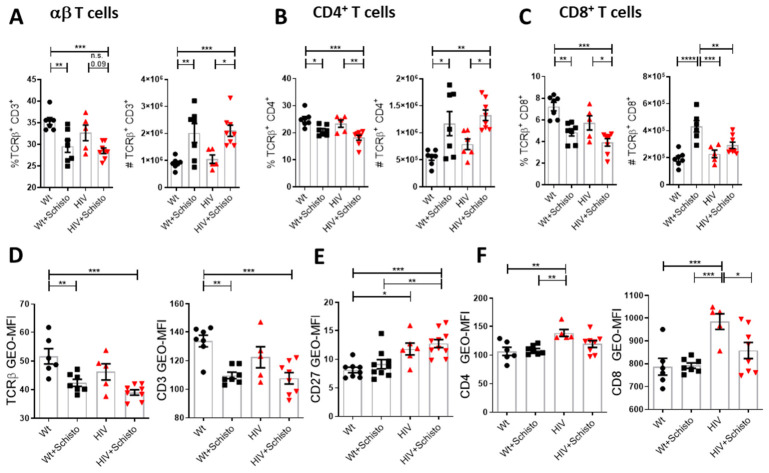
**Pulmonary αβ T cells in Wt and HIV mice exposed or unexposed to Schistosoma eggs.** Flow cytometric analysis of (**A**) αβ T lymphocytes (TCRβ^+^ CD3^+^), (**B**) TCRβ^+^ CD4^+^ and (**C**) TCRβ^+^ CD8^+^ T cells in the lungs of Wt and HIV mice unexposed or exposed (+Schisto) to *Schistosoma* eggs. Frequencies (%) and numbers (#) of cells for each population are shown. (**D**) Surface expression (GEO-MFI) of TCR (in TCRβ^+^ CD3^+^), (**E**) CD27 (in TCRδ^−^ CD3^+^), and (**F**) CD4 and CD8 (in TCRβ^+^ CD3^+^). Results are presented as mean ± SEM (n = 5–8). * *p* ≤ 0.05, ** *p* ≤ 0.01, *** *p* ≤ 0.001, **** *p* ≤ 0.0001 (one-way ANOVA Tukey).

Pulmonary lymphoid cell populations were characterised by flow cytometry as detailed in Supplementary Figure S2. Analysis of αβ T cells showed similar proportions and absolute counts in both untreated Wt and HIV mice. Following *Schistosoma* egg exposure, both groups exhibited a significant increase in absolute αβ T-cell numbers, despite a decrease in their relative frequency (Figure 4A).

Interestingly, surface TCR αβ expression was significantly reduced in Wt mice following egg administration, an effect that was less pronounced in HIV mice (Figure 4D). Furthermore, the activation marker CD27 was significantly higher in αβ T cells from untreated HIV mice compared to their Wt counterparts, with no further upregulation observed in either group after egg challenge (Figure 4E).

Within the αβ T-cell compartment, both CD4^+^ and CD8^+^ subsets were comparable in Wt and HIV mice but showed reduced proportions and increased cell numbers in both egg-treated groups (Figure 4B,C). However, the expansion of CD8^+^ cells was markedly less prominent in egg-exposed HIV mice than in Wt counterparts (Figure 4C). Notably, both CD4 and CD8 coreceptors were overexpressed in T cells from untreated HIV mice compared to Wt mice (Figure 4E). Following egg administration, coreceptor expression remained stable in Wt mice; in contrast, HIV mice exhibited a significant reduction in surface CD8 levels and a similar downward trend for CD4 (Figure 4E).

### 2.5. HIV Exposure Induced γδ T and NKT Cell Expansion Only in Combination with Schistosome Eggs, Reduced NK Cells and Had No Effect on B Lymphocytes

**Figure 5:**
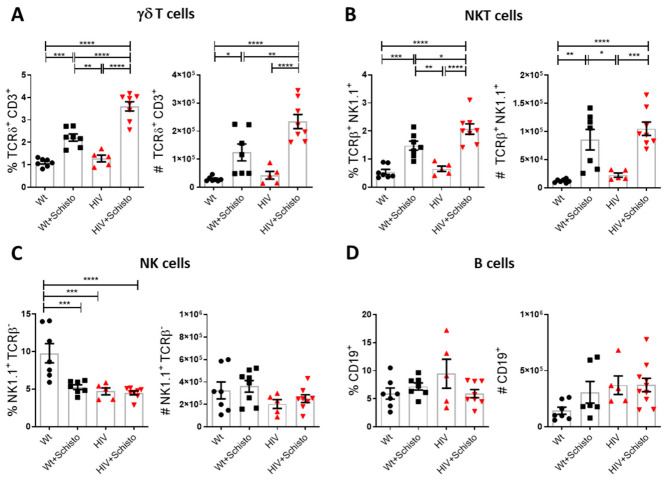
**Pulmonary γδ T, NKT, NK and B cells in Wt and HIV mice exposed or unexposed to *Schistosoma* eggs.** Flow cytometric analysis of (**A**) γδ T (TCRδ^+^ CD3^+^) cells, (**B**) NKT (TCRβ^+^ NK1.1^+^), (**C**) NK (NK1.1^+^ TCRβ^−^) and (**D**) B (CD19^+^) lymphocytes in the lungs of Wt and HIV mice unexposed or exposed (+Schisto) to *Schistosoma* eggs (n = 5–8). Frequencies (%) and numbers (#) are shown. * *p* ≤ 0.05, ** *p* ≤ 0.01, *** *p* ≤ 0.001, **** *p* ≤ 0.0001 (one-way ANOVA Tukey).

γδ T lymphocytes, which form a significant component of the T-cell repertoire alongside the αβ lineage, were identified by surface expression of the TCRγδ receptor. In untreated Wt and HIV mice, the frequency and absolute numbers of γδ T cells were comparable. Following schistosome egg administration, their abundance increased markedly in both groups, with a significantly greater expansion in HIV mice (Figure 5A).

NKT lymphocytes, characterised by the co-expression of T-cell (TCRβ) and NK-cell (NK1.1) markers (Supplementary Figure S2), were similarly represented in Wt and HIV mice. Both groups showed an increase in NKT cell numbers following egg exposure (Figure 5B), with a more robust response observed in the HIV group.

We next assessed NK (CD3^−^ TCRβ^−^ NK1.1^+^) cells. This lymphoid subset typically represents approximately 10% of mouse pulmonary lymphocytes [30]. As shown in Figure 5C, NK-cell frequencies were significantly lower in HIV mice compared to Wt controls, although absolute cell numbers did not differ significantly (despite a downward trend in the HIV group). In Wt mice, egg administration reduced the percentage of NK cells to levels comparable to those of untreated HIV mice, whereas the HIV group showed no further changes. Notably, absolute NK-cell numbers remained unchanged by schistosome egg administration in either group.

Finally, the B lymphocyte populations were assessed. Neither the frequency nor the absolute number of B cells differed between Wt and HIV mice, and no significant alterations were detected in either group after egg administration (Figure 5D).

## 3. Discussion

In the present study, we aimed to characterise the pulmonary immune cell landscape in mice exposed to HIV and *Schistosoma*, individually or in combination. To our knowledge, this is the first comprehensive profiling of pulmonary lymphoid and myeloid cells performed in the context of experimental or clinical HIV and *Schistosoma* co-morbidity.

Herein, we have confirmed the expression of viral proteins in the lungs of HIV-transgenic mice and that this expression was restricted to resident and/or infiltrating pulmonary leukocytes. In a previous study, we showed that schistosome egg administration resulted in a noticeable increase in the pulmonary leukocyte content in both HIV mice and Wt counterparts [20], most likely due to the infiltration of blood leukocytes. This is consistent with observations in the context of HIV infection, schistosomiasis [6,31] and mouse models of schistosome-induced PAH [6,31]. Embolisation of parasite eggs into the lungs leads to the formation of multicellular granulomas around trapped eggs. Although granulomas were smaller and less effective at destroying parasite eggs in HIV mice [20], they were morphologically similar to those of Wt mice and displayed a comparable cellular composition.

Pulmonary macrophages are a heterogeneous population of immune cells that perform a variety of specialised functions, including maintenance of pulmonary homoeostasis, removal of cellular debris, immune surveillance, microbial clearance, responses to infection and the resolution of inflammation [32] IMs are a macrophage subset located in the lung parenchyma, in contrast to AMs which reside in the airways. Both IMs and AMs showed a significant increase in egg-treated HIV mice compared with Wt counterparts or untreated HIV mice. Therefore, the increase in IMs in co-exposed mice suggests infiltration of the parenchyma by monocytes that later differentiate into IMs. Consistent with this, increased monocyte turnover was associated with the accumulation of pulmonary IMs in SIV-infected rhesus macaques [33]. Mouse IMs may exhibit some antigen-presenting capacity, as suggested by an earlier report [34]. Thus, in HIV mice, local expression of HIV proteins may promote monocyte recruitment to the lungs and their differentiation into IMs, which may act as both pro-inflammatory and antigen-presenting cells, thereby contributing to the modulation of subsequent T-cell responses. Our results are consistent with previous studies showing a role of increased IMs in pulmonary hypertension induced by *Schistosoma* [35] or hypoxia [36]. Activation of these IMs was associated with increased thrombospondin-1 and subsequent activation of TGF-β resulting in the pulmonary vascular remodelling characteristic of the disease [36,37]. Our present data also suggest that this immune population is exacerbated upon co-exposure with HIV proteins, which could contribute to the augmented pulmonary vascular remodelling and pulmonary arterial pressure found in animals co-exposed to *Schistosoma* and HIV [20]. Collectively, these findings demonstrate that prior HIV exposure primes the pulmonary environment, significantly enhancing the macrophage response to subsequent schistosome egg exposure, with a predominant effect on the IM compartment generated from local differentiation of recruited monocytes.

HIV and schistosome co-exposure resulted in a marked increase in patrolling-type monocytes. The described “patrolling” behaviour of migrating lung monocytes, as well as their localisation at the interface between the capillaries and the alveoli, suggested an immune surveillance function for these cells [38], although monocytic cells could also be pathogenic as inducers of fibrosis in the context of schistosomiasis [39]. Thus, lung patrolling monocytes could be involved in the exacerbation of fibrosis observed in schistosome egg-treated HIV mice [20]. Moreover, these cells were shown to actively infiltrate the small pulmonary arteries and differentiate into IMs, promoting vascular remodelling and the development of pulmonary hypertension after chronic hypoxia [40]. Thus, it is tempting to suggest that patrolling monocytes could also contribute to the exacerbated pulmonary arterial remodelling observed in mice co-exposed to HIV and *Schistosoma* [20]. Together, these data indicate that the interaction between HIV and *Schistosoma* exposure specifically modulates the patrolling monocyte pool without affecting recruitment of inflammatory monocytes.

Eosinophils and neutrophils participate in the immune response to multicellular parasites, and the former are involved in the granulomatous response to *S. mansoni* eggs [25]. Interestingly, the increase in these cell populations observed in Wt animals was not seen in HIV mice, suggesting that the presence of HIV proteins may alter the typical granulocytic response to parasite eggs in the lung.

Regarding pulmonary dendritic cells, the DC1 and DC2 subsets were equally represented in Wt and HIV mice and showed comparable increases in both groups upon schistosome exposure. These results indicate that, in a co-exposure context, schistosome eggs but not HIV, are the main stimulus for lung DC expansion; although with a greater impact on DC2 compared with DC1. In line with this, DC1 and DC2 dendritic cells have been recently shown to attenuate or promote, respectively, Th2-driven schistosome-induced PAH [41].

αβ T cells were actively recruited to the lung in response to parasite eggs, but because other leukocyte subsets predominated within the pulmonary infiltrate, their proportional representation appeared reduced. Moreover, analysis of the CD27 activation marker suggested that αβ T-cell activation is dysregulated in the presence of HIV proteins. Within the αβ T-cell compartment, CD4^+^ T cells have been directly implicated in PAH associated with schistosomiasis [22,42,43]. Pathogenesis in this context appears to depend on the inflammatory cascade triggered by parasite eggs, as part of the host immune response aimed to eliminating the parasite and controlling the infection [31], where Th2 cells producing IL-4 and IL-13 are thought to play a critical role at the onset of the egg-induced immune response [21]. In contrast, CD4^+^ T cells do not seems to play an important role in the development of HIV-related PAH, where disease progression appears to be more closely related to the duration of HIV infection [44]. Consistent with this, a recent study found that the number of alveolar CD4^+^ T cells in adults with HIV was comparable to that in uninfected controls [17]. Immunohistochemical studies of lung tissue sections from patients with idiopathic PAH showed a significant increase in CD4^+^ and CD8^+^ T cells in the adventitial space of the pulmonary vasculature [45], although their direct pathological relevance was not determined. In our model, HIV and schistosome co-exposed mice showed a marginally significant increase in lung CD4^+^ T cells compared to HIV untreated counterparts, but similar values to those of egg-treated Wt mice. This suggests that co-exposure could more critically impact the functional phenotype (e.g., the Th cytokine response [20]) than the abundance of lung CD4^+^ T cells, at least in a non-infectious context.

In contrast to CD4^+^ T cells, lung CD8^+^ T lymphocytes were significantly reduced in co-exposed mice, compared with egg-treated Wt mice, which is in contrast with natural HIV infection wherein lung CD8^+^ T cells are markedly expanded, particularly in the alveolar compartment [1,6]. This difference could be related to the non-infectious nature of our animal model. Alternatively, pulmonary HIV proteins could restrain the expansion of CD8^+^ T cells responding specifically to schistosome egg antigens. To any extent, the underlying mechanisms remain to be elucidated.

HIV and schistosome co-exposed mice showed increased abundance of NKT cells compared with egg-treated Wt counterparts. NKT cells play roles in anti-infection, anti-tumour, transplantation immunity, and autoimmune regulation. In addition, NKT cells can rapidly produce cytokines, modulate the Th1/Th2 balance, and stimulate or suppress immune responses in the lung [46]. The literature on NKT cells and their involvement in pulmonary vascular disease is scarce. In a study with *Schistosoma japonicum*-infected mice [47], the abundance of lung NKT cells was decreased compared to that of uninfected mice, which differs completely from our results with *S. mansoni* eggs, probably due again to the infectious vs. non-infectious approach used. In another study comparing PAH patients and controls, a tendency for increased blood NKT cells was observed in the former individuals, although this was not significant [48].

Similarly to NKT cells, γδ T cells were clearly increased in egg-treated HIV mice compared with individually exposed mice. γδ T cells are important mediators in pulmonary host defence that allow immediate responses to pathogens and are considered tissue-resident immune cells [49]. Lung-resident γδ T cells can be activated by antigens, pathogen-associated molecular patterns, damage-associated molecular patterns, activating receptor ligands or cytokine signalling. During viral infections, activated lung γδ T cells produce several types of cytokines, some of which inhibit virus replication, while others induce or inhibit lung inflammation [49].

γδ T cells have been implicated in various inflammatory diseases with distinct aetiology [50]. In HIV mice, but particularly in egg-treated ones, pulmonary γδ T cells could also act as important pro-inflammatory cells causing disease. In line with this, depletion of γδ T cells resulted in decreased inflammation and disease severity in a mouse model of viral lung disease [51]. Moreover, γδ T cells were detected in close proximity to pulmonary arteries in both healthy individuals and patients with idiopathic PAH, but at higher frequency in the latter [52]. It is worth noting that mice co-exposed to HIV proteins and schistosome eggs showed markedly increased IL-17A expression in γδ T cells [20]. This pro-inflammatory and profibrotic cytokine has been suggested to contribute to PAH development [53,54]. However, further studies are needed to clarify the potential pathogenic role of IL-17-expressing γδ T cells in pulmonary vascular diseases.

Although lung B lymphocytes were not significantly affected in HIV and schistosome co-exposed mice, they could be functionally relevant in the context of HIV proteins in the lung. Accordingly, in vitro models have shown that viral proteins such as Nef, which is expressed in HIV mice [55], can be accumulated by B lymphocytes [56] and indirectly promote polyclonal B-cell activation and increased CD4^+^ T-cell permissiveness to HIV infection. These effects were apparently related to Nef-induced secretion of pro-inflammatory cytokines by macrophages, which in turn upregulate the expression of costimulatory receptors on B lymphocytes [57].

Of interest, pulmonary NK cells in HIV mice were significantly decreased in frequency and showed a tendency for reduced numbers compared with Wt mice; and were unaffected by schistosome treatment. NK cells contribute to the clearance of HIV-infected cells during the acute phase and are key antiviral effectors of the innate immune system. Deficiencies of this cell type are also associated with an increased likelihood of HIV infection [58,59]. Notably, our findings in HIV mice are in line with the impaired pulmonary recruitment of NK cells reported in HIV infection [16].

Globally, schistosome egg administration caused a similar reduction in the lymphoid-to-myeloid ratio in Wt and HIV mice compared to untreated mice, indicating a predominance of myeloid cells in the leukocyte infiltrate in both groups, although myeloid cells were consistently more abundant in HIV than in Wt mice (data not shown). These results indicate that combined HIV and *Schistosoma* co-exposure could favour a biased recruitment of blood myeloid cells into the lung. This could be of pathological relevance as increased myeloid cells have been associated with pulmonary hypertension in HIV [60] and fibrosis [61]. In addition, a lymphoid-to-myeloid ratio profile similar to that of egg-treated HIV mice has been reported for PAH [62].

In this study, we have shown that HIV-transgenic mice, which harbour a replication-deficient HIV-1 proviral genome, express HIV proteins in pulmonary immune cells, resembling people with HIV receiving ART. In these individuals, ART blunted viral replication but it was ineffective at eliminating pulmonary HIV latent reservoirs, which in turn are associated with the persistence of HIV proteins in the lungs [1,6]. Also of note, ART normalised the numbers of pulmonary αβ T cells in people with HIV [6], in agreement with our data showing no significant alterations in CD4^+^ and CD8^+^ T-cell abundance in HIV-transgenic mice. Thus, our co-exposure mouse model would specifically mimic ART-treated, but not ART-naïve, individuals with HIV infection who subsequently acquire *Schistosoma* infection. Unfortunately, the paucity of information on HIV and *Schistosoma* co-infection [63,64] precludes a deeper comparative analysis between our co-exposure model and natural co-infection.

Although the precise mechanisms underlying the differential effects of HIV and *Schistosoma* co-exposure on lung immune cells remain to be elucidated, it could be hypothesised that HIV proteins intrinsically expressed by lung immune cells could impair their homeostasis or functionality. Alternatively, or additionally, viral proteins could be released locally by these cells (either actively or after cell death) and affect other immune cells or those of the lung vasculature [65,66,67]. Thus, the persistence of HIV proteins in the lung, together with the inflammatory effects of embolised parasite eggs, could explain their unique impact on the pulmonary immune cell landscape and the endothelial remodelling and dysfunction [20] observed in co-exposed mice.

## 4. Materials and Methods

### 4.1. Animal Samples

We used lung samples derived from the mice described in our previous report [20]. Samples from four groups of mice were evaluated in parallel: wild-type (Wt, n = 7), *Schistosoma* egg-treated Wt (Wt + Schisto, n = 7), HIV (n = 5), and egg-treated HIV (HIV + Schisto, n = 8). HIV (Tg26)-transgenic mice carry a replication-deficient HIV-1 proviral genome that encodes the Env protein and the accessory genes *tat*, *nef*, *rev*, *vif*, *vpr*, and *vpu*. The deletion of a significant portion of the *gag-pol* region ensures the model is non-infectious while allowing for systemic transgene expression [55]. To model *Schistosoma* exposure, mice were sensitised and subsequently challenged with inactivated *S. mansoni* eggs to induce pulmonary embolization, following previously established protocols [20,21]. Animal procedures were carried out according to the Spanish Royal Decree 1201/2005 and 53/2013 on the Care and Use of Laboratory Animals and ethically reviewed by the institutional Ethical Committees of the Universidad Complutense de Madrid (Madrid, Spain) and approved by the regional Committee for Laboratory Animals Welfare (Comunidad de Madrid, Ref. number PROEXO-003/18).

### 4.2. Immunofluorescence in Lung Sections

Immunostaining was performed on paraffin-embedded tissue. Lung sections (3-μm) were dewaxed and rehydrated. To retrieve antigens, slides were then heated for 20 min at 95 °C in citrate buffer (10 mM, pH 6.0) (Thermofisher, Inc., Waltham, MA, USA, Ref. 005000) in a cooker, with 10 min of cooling at room temperature. After washing with phosphate-buffered saline (PBS), sections were blocked for 1 h at room temperature with BSA 5%. Blocked sections were incubated overnight at 4 °C with combinations of primary antibodies (Supplementary Table S1) followed by washing and incubation for 1 h at RT with matched fluorophore-conjugated secondary antibodies (Supplementary Table S2). For triple and quadruple staining, the same protocol was repeated for primary and secondary antibodies on consecutive days. Nuclei were stained with DAPI (Thermo Scientific, Inc., Waltham, MA, USA, Ref. 62248), and slides were mounted using a water-based mounting medium (Fisher Scientific).

### 4.3. Isolation of Lung Leukocytes

Mouse lungs were perfused via the right ventricle with PBS and processed for flow cytometry. Left lung lobes were finely minced and digested in RPMI containing 1 mg/mL Liberase TM (Sigma-Aldrich Quimica, Madrid, Spain) for 30 min at 37 °C. The reaction was stopped by adding EDTA, followed by incubation for 2 min. Subsequently, 1 mL of RPMI was added, and tissues were mechanically dissociated by vigorous pipetting (at least 50×) using a 1 mL Pasteur pipette. The resulting cell suspension was filtered through a 40 µm cell strainer (Corning, New York, NY, USA) and centrifuged at 300× *g* for 10 min. The supernatant was discarded, and the cell pellet was resuspended in 1 mL ACK lysing buffer (Gibco, Thermo Fisher Scientific, Inc., Waltham, MA, USA) for 10 min to lyse red blood cells. Lysis was stopped by adding 14 mL RPMI, followed by centrifugation at 300× *g* for 10 min. The final cell pellet was resuspended in RPMI. Cell counts were determined using a Neubauer chamber with trypan blue exclusion to assess viability.

### 4.4. Cell Staining and Flow Cytometry

For flow cytometry analysis, cells were resuspended in FACS buffer (PBS containing 5% BSA and 0.1% sodium azide). Samples were adjusted to 5 × 10^5^ cells/100 µL of FACS buffer. Following Fc receptor blocking with anti-CD16/CD32 antibodies (BD Biosciences, San Jose, CA, USA), cells were stained for extracellular leukocyte markers using a combination of fluorochrome-conjugated antibodies at a concentration of 1 µg/mL, and incubated for 30 min at 4 °C in the dark. Cells were then centrifuged, the supernatant discarded, washed twice and fixed with 1% BD Cell Fix (BD Biosciences, San Jose, CA, USA). Finally, cells were washed and prepared for analysis. Data were acquired using a BD FACSCalibur flow cytometer and analysed with FlowJo v10 software (BD Biosciences, FlowJo, LLC, Ashland, OR, USA), according to the gating strategies depicted in Supplementary Figures S1 and S2.

### 4.5. Statistical Analysis

Data are expressed as mean ± SEM.; n indicates the number of animals from which the samples were processed in each group. Each data point shown is an individual mouse and therefore constitutes a biological replicate. Statistical analysis was performed using Student’s *t* test and one-way ANOVA (for normally distributed data) followed by a Tukey post hoc test. Two-way ANOVA and the Bonferroni multiple comparison test was used to compare dose–response curves. Differences were considered statistically significant when the *p* value was less than 0.05.

## 5. Conclusions

In conclusion, this study demonstrates for the first time that combined pulmonary exposure to HIV and *Schistosoma* affects the local immune cell landscape in a manner distinct from individual exposures. The implications of these findings could be significantly helpful in understanding the pathogenesis of pulmonary vascular remodelling and consequently the development of PAH. This may be valuable for the future development of novel therapeutic approaches for pulmonary diseases associated with HIV infection and its co-morbidities, such as schistosomiasis.

## Data Availability

The original contributions presented in this study are included in the article. Further inquiries can be directed to the corresponding authors.

## Supplementary Materials

The following supporting information can be downloaded at https://www.mdpi.com/article/10.3390/ijms27125426/s1.
