# Supplementary material for: Pulmonary Immune Cell Landscape Altered by Exposure to HIV, Schistosoma and Their Combination
Source: Int J Mol Sci. 2026 Jun 16;27(12):5426. doi: 10.3390/ijms27125426 (PMC13300048; doi:10.3390/ijms27125426)
Supplement: Supplementary file 1 [file ijms-27-05426-s001.zip › ijms-4322390-supplementary.pdf]

Supplementary material

Pulmonary Immune Cell Landscape Altered by Exposure to HIV, *Schistosoma* and Their Combination

Daniel Morales-Cano [1,2,*,†], Sandra Medrano-Garcia [3,4,†], Bianca Barreira [1,2], Ana Hernández-García [1,2], Rahul Kumar [5], Brian B. Graham [6], Rajkumar Savai [4,7], Soni Savai Pullamsetti [4,7], Francisco Perez-Vizcaino [1,2], Ghazwan Butrous [8], Angel Cogolludo [1,2,*,†] and Edgar Fernández-Malavé [3,†]

[1] Department of Pharmacology and Toxicology, School of Medicine, Universidad Complutense de Madrid, Instituto de Investigación Sanitaria Gregorio Marañón, 28040 Madrid, Spain; biancabarreira@med.ucm.es (B.B.); anaher10@ucm.es (A.H.-G.); fperez@med.ucm.es (F.P.-V.)
[2] Centro de Investigación Biomédica en Red Enfermedades Respiratorias, (CIBERES), 28029 Madrid, Spain
[3] Department of Immunology, Ophthalmology and ENT, School of Medicine, Universidad Complutense de Madrid, Instituto de Investigación Sanitaria Hospital 12 de Octubre (imas12), 28040 Madrid, Spain; samedran@ucm.es (S.M.-G.); edfernan@med.ucm.es (E.F.-M.)
[4] Institute for Lung Health (ILH), Justus Liebig University, 35305 Giessen, Germany; rajkumar.savai@mpi-bn.mpg.de (R.S.); soni.pullamsetti@mpi-bn.mpg.de (S.S.P.)
[5] Department of Translational Immunology, Genentech, Inc., 1 DNA Way, South San Francisco, CA 94080, USA; kumar.rahul.rk1@gene.com
[6] Department of Medicine, University of California, San Francisco, CA 94143, USA; brian.graham@ucsf.edu
[7] Max Planck Institute for Heart and Lung Research, Member of the German Center for Lung Research (DZL), Member of the Cardio-Pulmonary Institute (CPI), 61231 Bad Nauheim, Germany
[8] Cardiopulmonary Sciences, University of Kent Canterbury, Kent CT2 7NZ, UK; g.butrous@kent.ac.uk
* Correspondence: damora02@ucm.es (D.M.-C.); acogolludo@med.ucm.es (A.C.)
† These authors contributed equally to this work.

Table of contents

Supplementary Figure S1. Flow cytometry gating strategy for identification of pulmonary myeloid cells (page 3).

Supplementary Figure S2. Flow cytometry gating strategy for identification of pulmonary lymphoid cells (page 4)

Supplementary Table S1. Primary antibodies for immunostaining of lung sections (page 5).

Supplementary Table S2. Secondary antibodies for immunostaining of lung sections (page 5).

Supplementary Table S3. Antibodies for flow cytometry (page 6).

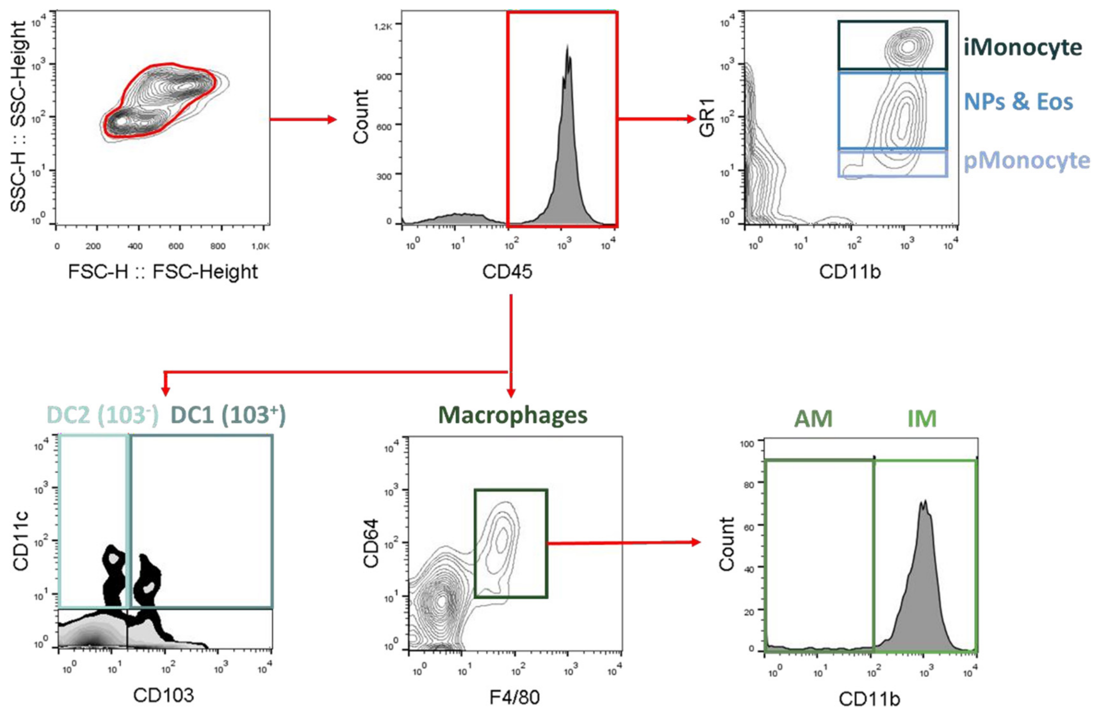

Supplementary Figure S1. Flow cytometry gating strategy for identification of pulmonary myeloid cells. iMonocyte: inflammatory monocyte; pMonocyte: patrolling monocyte; NPs: neutrophil; Eos: eosinophil; DC: dendritic cell; AM: alveolar macrophage; IM: interstitial macrophage.

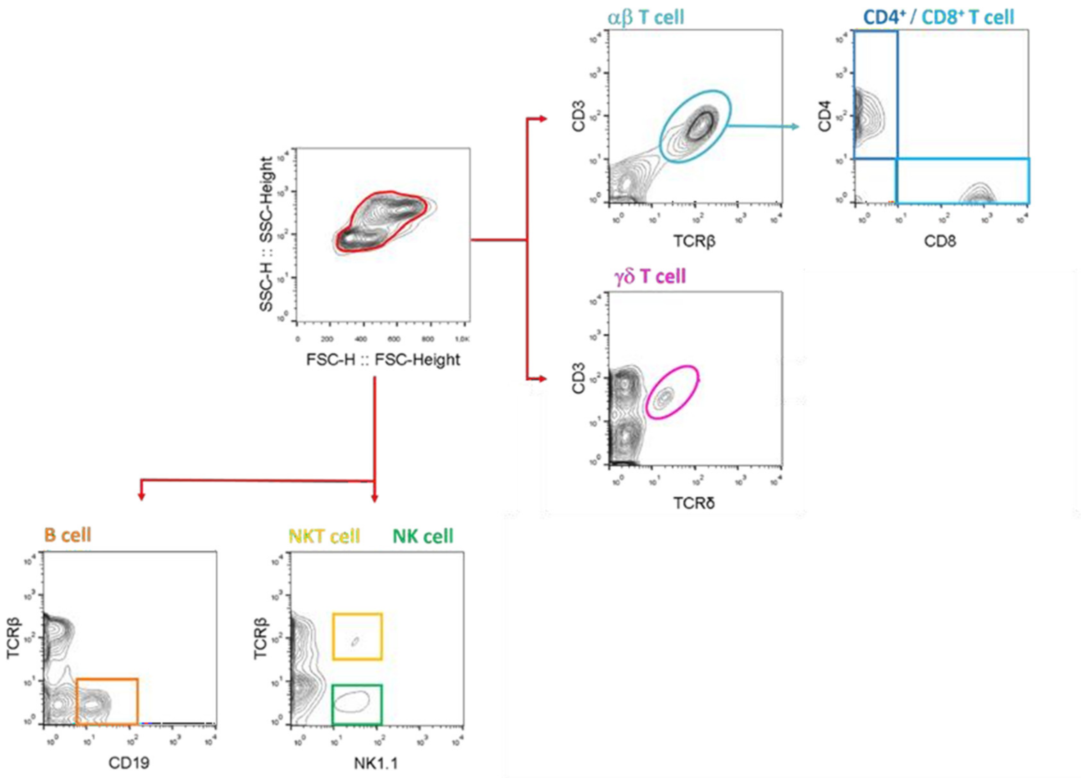

Supplementary Figure S 2. Flow cytometry gating strategy for identification of pulmonary lymphoid cells.

Supplementary Table S1. Primary antibodies for immunostaining of lung sections.

Antibody	Reactivity	Host	Source	Reference
CD45	mouse	rabbit	Sigma	SAB4502541-100U6
CD45	mouse	rat	Novus	NB10077417SS
F4/80	mouse	rabbit	Cell Signalling	70076s
NEF (HIV)	HIV	mouse	Abcam	ab42358

Supplementary Table S2. Secondary antibodies for immunostaining of lung sections.

Antibody	Conjugate	Source
Goat Anti-Rabbit	Alexa Fluor® 555	Invitrogen
Goat Anti-Mouse	Alexa Fluor® 555	Abcam
Goat Anti-Rat	Alexa Fluor® 594	Invitrogen

Supplementary Table S3. Antibodies used for flow cytometry

Antibody	Clone	Source
CD11b	M1/70	E-BioScience
CD11c	HL3	BD Pharmingen
CD19	ID3	E-BioScience
CD27	L67F9	E-BioScience
CD3ε	145-2C11	BD Pharmingen
CD4	GK1.5	E-BioScience
CD45	30-F11	E-BioScience
CD64	X54.5/7.1	BD Pharmingen
CD8	H35-17.2	E-BioScience
F4/80	T452342	BD Pharmingen
NK1.1	PK136	BD Pharmingen
TCRβ	H57597	BD Pharmingen
TCRδ	GL3	BD Pharmingen
